# The complex remuneration of human resources for health in low-income settings: policy implications and a research agenda for designing effective financial incentives

**DOI:** 10.1186/s12960-015-0058-7

**Published:** 2015-07-28

**Authors:** Maria Paola Bertone, Sophie Witter

**Affiliations:** ReBUILD consortium, Department of Global Health and Development, London School of Hygiene and Tropical Medicine, 15-17 Tavistock Place, London, WC1H 9SH UK; ReBUILD Consortium, IIHD, Queen Margaret University, Edinburgh, UK

**Keywords:** Human resources for health, Low-income countries, Financial incentives, Complex remuneration, Pay

## Abstract

**Background:**

Human resources for health represent an essential component of health systems and play a key role to accelerate progress towards universal health coverage. Many countries in sub-Saharan Africa face challenges regarding the availability, distribution and performance of health workers, which could be in part addressed by providing effective financial incentives.

**Methods:**

Based on an overview of the existing literature, the paper highlights the gaps in the existing research in low-income countries exploring the different components of health workers’ incomes. It then proposes a novel approach to the analysis of financial incentives and delineates a research agenda, which could contribute to shed light on this topic.

**Findings:**

The article finds that, while there is ample research that investigates separately each of the incomes health workers may earn (for example, salary, fee-for-service payments, informal incomes, “top-ups” and per diems, dual practice and non-health activities), there is a dearth of studies which look at the health workers’ “complex remuneration”, that is, the whole of the financial incentives available. Little research exists which analyses simultaneously all revenues of health workers, quantifies the overall remuneration and explores its complexity, its multiple components and their features, as well as the possible interaction between income components. However, such a comprehensive approach is essential to fully comprehend health workers’ incentives, by investigating the *causes* (at individual and system level) of the fragmentation in the income structure and the variability in income levels, as well as the *consequences* of the “complex remuneration” on motivation and performance. This proposition has important policy implications in terms of devising effective incentive packages as it calls for an active consideration of the role that “complex remuneration” plays in determining recruitment, retention and motivation patterns, as well as, more broadly, the performance of health systems.

**Conclusions:**

This paper argues that research focusing on the health workers’ “complex remuneration” is critical to address some of the most challenging issues affecting human resources for health. An empirical research agenda is proposed to fill the gap in our understanding.

## Introduction

Human resources for health (HRH) represent an essential component for the functioning of health systems [1] and play a key role to accelerate progress towards universal health coverage [2-4]. However, many low-income countries face challenges with reference to availability of health workers, which is the supply of qualified workers; distribution, that is, recruitment and retention of health workers where they are needed; and performance, which is the productivity and the quality of their work [5]. Theoretical research has explored the factors underlying health workers’ motivation [6,7], and empirical studies and systematic reviews have looked extensively at the factors (or combination of factors) that improve recruitment and retention and enhance motivation. They find that the possible strategies are multiple and include financial benefits (pay and other benefits, such as pension, insurance, accommodation), indirect benefits (subsidized transport, food, child care) and non-financial benefits (access to training, social recognition, supervision, etc.) [6]. While some studies suggest that a payment or salary is an essential precondition for the motivation of health workers [8], others argue that non-financial incentives can be highly effective, especially for the attraction and retention in rural areas [9,10]. Yet, most scholars agree that “bundles of interventions” addressing multiple factors and combining financial and non-financial incentives work better than interventions limited to one single type of incentive [10-13].

In this article, we focus exclusively on the financial component of incentive packages provided to health workers and specifically on health workers’ remunerations and revenues. We argue that, while there are various bodies of literature in health economics and health policy and systems research in low-income settings that look separately at some of the incomes and examine the effect of each on health workers’ motivation, there has been a general lack of attention to the “complex remuneration” of health workers in a comprehensive way, including the whole of the financial incentives and revenue sources available, and to how the different incomes may interact. Indeed, in most low-income settings, the remuneration of health workers is not composed of a single type of payment but includes combinations of payment mechanisms, which differ by source of funding, contract agreements, features such as regularity and inclusion of “deferred” compensation (pensions), and task requirements. The thorough understanding of the entire remuneration of health workers and each of its components, as well as the acknowledgement of its complexity by researchers and policy-makers, is extremely relevant in order to devise effective overall incentive packages.

This article first describes the perspectives taken by the existing economics and health system literature on HRH remuneration in low-income settings and then introduces a broader approach to the study of health workers’ financial incentive environment which has thus far been little explored. The article concludes with some reflections on the policy and research implications of our proposition.

### Perspectives on HRH remuneration in the existing literature

The remuneration of workers, along with the related issues of incentives and motivation, have been discussed and analysed by different bodies of literature, both theoretical and empirical – the latter focused mostly on high-income settings. One of the most broadly adopted conceptual frameworks to explore the role of remuneration and incentives in defining behaviour in work relationships is “agency theory”. Agency theory studies a setting where a “principal” delegates authority to an “agent” who is working on behalf of the principal to perform a task. Because of her imperfect information on the agent’s effort and skills, the principal devises contracts that include rewards or sanctions (usually in the form of a financial remuneration) to elicit the desired behaviour [14]. Mainstream economic models predict (under a series of assumptions, including that of “materialistic self-interest” of individuals) which type of incentives should be included in the “ideal” contract (for example, piece-rate payments, fixed salary or a mix), applying concepts from institutional economics, such as “high-powered” and “low-powered” incentives [15,16]. Most recently, economic work began expanding the microfoundations of agency theory to allow for non-rational behaviour and social preferences of individuals. Going beyond revenue and effort as the sole explanatory factors, behavioural models add psychological factors to the agent’s utility maximization problem. These factors include inequality aversion, teamwork, and professional and identity norms and have been explored by theoretical and empirical literature [16,17]. The complex process of the motivation of health workers and the role of factors beyond the financial remuneration (including intrinsic determinants, the work context and the broader societal culture) has been acknowledged widely, and their study crosses many disciplinary boundaries, including economics, psychology, organizational development, human resource management and sociology [7]. While we do not aim to present a systematic review of the vast and varied research available, in order to provide a basis for our argument, we focus here on a selected portion of the health economics and health policy and system research literature, which looks at health workers’ financial incentives in low-income settings.

In some of the health economics literature, health workers’ remuneration arrangements are seen as “provider payment mechanisms” targeting individuals, under which health workers could be receiving a salary, a fee-for-service payment, capitation or a payment based on their performance. The empirical literature on the incentives created by the different types of payment has been reviewed by many [18-23]. Its focus is found to be predominantly on high-income countries, as fewer, if any, economic analyses have been performed in low- and middle-income settings. It is generally implied that health workers in low- and middle-income countries receive a salary for their public sector activities and are paid a fee-for-service for their private ones [24].

Yet, the actual composition of their remuneration is much more complex, as shown by empirical work carried out in low-income countries. Indeed, these studies point out to the fact that the categories of income sources defined in the health economics literature, and their mutual exclusivity, are less relevant in those contexts. Roenen et al. [25] identified 8 categories (and 28 sub-categories) of medical and non-medical income-generating activities, ranging from agro-pastoral and commercial work to secondary jobs within the public sector (for example, teaching), per diems and premiums, private practice and informal incomes, such as gifts from patients, and appropriation of public resources. In practice, very few studies have quantified the overall remuneration of health workers presenting information on each of these components. These papers generally overlook the complex remuneration and its potential consequences on health workers’ motivation as an issue in itself and focus on other questions, namely the impact of changes on HRH (including their income) with the introduction of a fee exemption scheme [26,27], the adequacy of health workers’ income and the fiscal and macroeconomic aspects of health workers’ remuneration [28] or the strategic tools available for policy-makers to control health workers’ behaviour [29].

Other research has focused on the income of health workers in order to explore their individual “financial coping strategies”, that is, the ways workers deal with their financial needs. This body of work aims at assessing the adequacy of the public health workforce salary in comparison to alternative work [30] or at investigating the consequences of the coping strategies on the public health system, in order to devise policies to reduce the need to adopt such strategies [31-33], or to put in place performance-based bonuses sufficiently high to incentivize health workers and compensate their increased efforts [34]. Along similar lines, a diverse body of literature focuses on those sources of income that are considered “informal” or downright “illegal”, looking at dual practice and moonlighting [35-39] as well as activities within facilities, such as charging under-the-table fees and selling pharmaceuticals [31,40,41]. The main objective of these studies is to attempt the, obviously difficult, assessment of the level of each of those incomes and to discuss their implications in terms of the distortions they can create on the main public job (for example, competition for time and absenteeism) and on the governance of the health system.

Other studies have focused on the widespread practice of external organizations of paying salary supplementations (“top-ups”) and “per diems” to health workers. Although meant to reimburse real expenses, per diems are usually paid well beyond the level necessary to cover the actual costs on the activities concerned, and they have attracted much criticisms because of the distortions and abuse to which they are subject and the increase in corruption that they may cause [42-46]. From their perspective, international donors are aware of the critical role they play by providing unofficial supplements to health workers’ salaries. The open discussion held at the 1998 International Conference in Lisbon [47-51] is particularly useful in this respect. However, little empirical work exists to measure this impact and its consequences. An exception is the body of literature on the “system-wide effects” of Global Health Initiatives which looks at incentives created by such programmes when they include remuneration to health workers. It is found that, indeed, Global Health Initiatives have contributed substantially to salary top-ups and per diems paid mostly for in-service training [52-55]. The main concern is the evidence of their consequences in terms of “brain drain” from public posts to NGOs and bilateral agencies [56-58], as well as of additional workload, distortion from routine activities and absenteeism in public (usually policy-making) positions [59-61].

With the widespread introduction, in many countries of sub-Saharan Africa, of performance-based financing (PBF) schemes, which often entail a bonus for staff, another payment is available for health workers. The core concept of PBF schemes is to make use of incentives in order to promote better health service coverage and results, by linking financial incentives to desired outputs and encouraging increased effort [62,63]. Critics of the approach have suggested that PBF schemes may promote “gaming” practices, distortions in service delivery in favour of services included in the scheme and crowding-out the intrinsic motivation of health workers [64,65]. Empirical evidence from low-income countries on how PBF schemes affect health workers’ motivation is still limited, but some preliminary results are available. The analysis of an early PBF scheme in Rwanda showed the sharp increase in staff productivity [66]. In Benin, with the introduction of two pilot PBF projects, health workers report being more professional and respectful of standards, but their motivation is limited by the perceived unfairness in bonus distribution [67]. A quantitative study in the Haut-Katanga region of DR Congo finds that the PBF scheme led to more effort from health workers without crowding-out of non-targeted services, staff conflicts, gaming or free-riding. However, the scheme, because of the effect it had of reducing the overall remuneration of health workers, led to a decrease in their intrinsic motivation [68].

Most recently, research has explored HRH incentive issues from a labour market perspective [5,69-71]. This body of work argues that to address issues of maldistribution, low retention and poor performance of health workers, a shift of focus from health workforce planning to other factors, such as labour market dynamics and the behavioural responses and individual preferences, is needed. The proposed approach looks at the national and international market for health workers and at the competing alternatives to public employment, such as private practice and migration to other countries. Although this work allows for the possibility of dual practice, the “price” considered for the health labour market is represented by the “wage rate” and there is limited attention to the existence and consequences of simultaneous, multiple incomes.

### The complex remuneration of health workers: policy implications

Although most of the studies reviewed above describe the remuneration of health workers or rather some components of it, with few exceptions [26,27,29,34], they do not adopt a comprehensive approach reflecting the overall financial incentives and encompassing all incomes available. Importantly, however, taken as a whole, this literature points out to the existence and relevance of the phenomenon that we call “complex remuneration”, which is the fact that health workers earn their living from a variety of sources and activities. We believe that a broader and more integrated understanding of the financial incentive environment available for health workers is necessary and of high-policy relevance. While the work done so far tends to reflect on different incomes separately, further research is needed on all these elements simultaneously. Such research would allow describing and quantifying the overall income and each of its components, including their relative importance and their variation across individuals. Moreover, it could explore hypotheses (i) relating to the *causes* of differences in income structure and levels between individuals, as well as within and across countries and their linkages with the fragmentation of the health systems, and (ii) on the potential interactions between incomes, the incentives created by the complex remuneration structure and their *consequences* on health workers’ motivation, behaviour and, more broadly, on the health system performance.

From a policy perspective, the issues raised by the complex remuneration pose numerous new challenges in order to establish rational and aligned financial incentive packages to recruit, retain and motivate the health workforce. While some guidance already exists for devising health workers’ incentives and addressing attraction and retention issues [72,73], under the assumption that the overall income of health workers depends on various and interacting factors beyond the ones that are usually considered, such as individual education and type of posting, the design of financial incentives becomes increasingly difficult. Other elements and factors should now be considered, such as the opportunities for external payments or for medical and non-medical activities beyond the main employment. The need for broader consideration of revenue sources beyond the salary is valid for any type of financial payment or strategy that is to be introduced, from rural allowances to PBF bonuses which have to take into consideration the overall income of health workers in order to be sufficient to produce an impact on motivation and, at the same time, to avoid the “blurred” and ineffective incentives created by the accumulation of various payments [67]. Moreover, the complex remuneration of health workers in many low-income settings presents specific challenges in that multiple payers and lines of accountability exist, with potentially clashing agendas that influence the activities health workers perform. This is different from multiple payment systems in high-income countries, where a single principal is more able to align incentives [74].

Policy-makers and their partners at the national level are called to pay increased attention to the wider financial incentive environment, as well as to engage in reflections to inform HRH reforms going beyond the issue of salaries and governmental allowances. The policy-making processes must be supported by the collection of relevant data (including qualitative) and the creation of an information base on these issues. Additionally, the policy debate at the central level should be framed within the broader macrolevel context of HRH incentives (which includes issues such as caps on total wage bills) and should take into consideration how microlevel strategies for the motivation of health workers can be affected and at times constrained by macrolevel conditions. Finally, beyond the national level, it has to be acknowledged that some of the incomes are subject to and influenced by local-level dynamics (Bertone MP, Witter S: An exploration of the political economy dynamics shaping health worker incentives in three districts in Sierra Leone, submitted). For example, private practice is usually more widespread in urban areas rather than in rural ones, and depending on the geographical distribution of donors and NGOs, per diems, top-ups or other payments may be more common or higher in certain areas of a country than in others.

### An agenda for research on the health workers’ complex remuneration

Further research is needed in order to support policy design and decisions, tailored to the specificity of the contexts. An innovative agenda of health policy and systems research would require exploring the complex remuneration of health workers and refining the necessary tools to capture it. The hypotheses that motivate such research agenda are multiple, and the main issues and research questions that could be explored under this proposition are described below and in Table 1.

**Table 1:** **Main hypotheses of the proposed research agenda on HRH complex remuneration and possible research questions**

**Main issues/hypotheses**	**Possible research questions**
Complexity/fragmentation of income sources	• What are the different incomes available for health workers and the level and relative importance of each income?
	• To which type of health workers is each source of income available – including health worker characteristics at the individual level (such as age, gender, level of education, years in the health sector and role within the facility) and at the facility level (type of facility, rural/urban, size, location within the country, etc.)?
	• What are the individual- and facility-level determinants that define the level (amount) of each income received/earned?
	• How do the different incomes interact with each other? Are certain incomes used as a substitute for the lack/low level of others or rather as complements?
	• How are the different incomes used by health workers?
Consequences of the complex remuneration	• What are the features of the different revenues (for example, present vs. deferred and stable vs. irregular, performance-based vs. fixed), and how do these affect motivation and performance?
	• How do health workers perceive their incomes, in terms of fairness, of being sufficient to motivate them, of transparency on what influences them, etc.? How do these perceptions affect their motivation and performance?
	• What are the consequences of the income fragmentation on the motivation and performance? (for example, does the accumulation of payments lead to “blurriness” and decrease effectiveness of incentives?)
	• How do different incomes and their fragmentation affect the time spent on different activities, levels of absenteeism and accountability links to different payers?
Differences in total income across health workers of the same cadre	• What is the measure of differences of income across similar health workers? (that is, same cadre/level of education and type of post and role within facility)
	• What are the drivers at the individual and facility level of these differences?
	• What are the consequences of the inequalities of total income? Are they justifiable and have a motivating effect (for example, incentivizing rural workers)? Or do they cause unacceptable inequalities and hamper availability, retention and distribution, as well as motivation (for example, urban workers or workers in some areas have more opportunities to earn some revenues from private practice or donors’ support)?
Differences within countries	• Are there income differences (both overall and for each component) between health workers in different areas of the same country?
	• What are the causes of these differences? (for example, rural/urban divide, different socio-economic contexts, historical legacies, political economy dynamics at local level and presence of external actors)
Differences across countries	• Do health workers in some countries have more complex incomes than in others? Why? (for example, different health system architecture and health system fragmentation, role of private sector, existence of free health care policies, level of health funding and fragile/post-conflict settings)
	• Are individual differences for similar health workers more important in some countries than others? Why?
Designing financial incentive packages	• Which tools and methods are needed to produce context-specific evidence in order to design rational and effective incentive packages for health workers?
	• What is the role of governments and their development partners in reducing inequalities and fragmentation of health workers’ income?
	• What are the policy implications of complex remuneration (for example, its effects on policy options and effectiveness), and what are the options for addressing it?
	• How are individual-level strategies for the motivation of health workers affected and constrained by macrolevel conditions (for example, wage bills caps)?

First, a description of the level of fragmentation and complexity of the overall income in a country as well as across countries would be extremely useful to explore what are the revenues available to health workers, including their absolute and relative levels. Such work could focus on the causes and determinants of the incomes, looking at variables at the individual, facility and geographical level (for example, which health workers receive each income? Who receives more for each income?). Furthermore, hypotheses on the consequences of the fragmented and complex remuneration should also be investigated. Different sources, levels and features of each revenue – such as predictability, regularity, link with deferred compensation (that is, pension and increases with career progression), type of contract (for example, performance-based or fixed), source of payment and tasks required (for example, routine or disease-specific and within facility or outside) – may play a key role in affecting health workers’ behaviour and motivation and therefore performance, in different ways. Moreover, specific requirements related to income component may affect time spent by health workers on different activities (for example, top-ups for disease/service-specific work may increase time spent on those) and the presence at work (for example, incomes earned outside of the facility, such as per diems or non-health-related work). These issues could be further researched with a comprehensive approach. Other key issues are the individual perceptions about the sufficiency, fairness and transparency in the allocation of the revenues [75,76], as well as social and cultural views over certain incomes, all of which are likely to affect the motivation of health workers. For example, it is possible that, in some contexts, the government salary may be a relatively limited and unreliable source of income but perceived as extremely important either because it is linked to pension benefits and job security [76,77] or because health workers assign a significance beyond its immediate monetary value. In the DR Congo, Fox et al. found that this was the case as receiving a salary is seen as a social recognition of the health worker’s role [78]. Similarly, some revenues may be low in absolute terms but they could enable access to other “goods” (such as training or social status) or, because of their regularity and predictability, could act as income “stabilizers” and therefore be considered important by health workers. A further unexplored hypothesis relates to the potential interaction between income components. If we consider the possibility of earning simultaneously different revenues, some incomes could play a role either as a substitute for meagre official payments or as a complement to those, even when their level is sufficient. For example, are revenues for activities outside of the health sector, such as agriculture or business, earned to “make ends meet”, or are those incomes available only to workers who earn enough from other sources and are therefore able to make further investments?

Turning to the overall revenue, it is likely that, given the fragmentation, the total income may differ for health workers even within the same cadre and level of education. In this case, it will be important to assess the level of income variability and investigate its causes. These differences could be a used as a motivation tool by incentivizing health workers to work in rural areas or ensuring their career progression, especially if remuneration is transparent and fragmentation reduced. On the other hand, these differences may be a possible source of inequity between individuals and demotivation. Research could explore by which income component(s) differences in total income are driven and/or whether these differences are related to characteristics at the individual level, such as gender, or at the facility level (rural or urban location) or at the geographical level (different districts or provinces). Based on answers to such questions, it is possible to reflect on the policy relevance of the income differences: are differences justifiable and used to address availability, distribution and retention issues, or do they cause unacceptable inequalities? Are inequalities avoidable and policy-amenable? If so, what are the policy tools to address them? For example, a study of doctors in Viet Nam found that the difference in opportunities for financial revenues between areas of posting favoured those in urban areas. The fact that these differences mostly originated outside official pay channels and were of large magnitude presented a considerable policy challenge to address distribution imbalances [29].

Another set of hypotheses concerns the difference in the complexity of income composition that there may be within and across countries. A question in this case is whether the fragmentation of revenue sources and the variation of total income for similar health workers have local determinants and/or mirror the fragmentation of the health system and increase in contexts where numerous (external) actors are involved, such as in fragile states/regions or where private practice is widespread.

The call for a novel approach focused on the overall remuneration and including sources of income that are both formal and informal also requires refining existing methods to elicit such information [79], as well as testing and defining new ones. Informal revenues are extremely difficult and perhaps impossible to precisely calculate because of the reticence of the health workers to openly declare them and the absence of records. However, some potentially useful techniques that allow rough estimates have been explored [34,40]. Revenues that are not harmonized but vary for each health worker, such as top-ups and per diems, are difficult to collect other than through individual surveys, given the difficulty of obtaining disaggregated data from donors’ databases. Mixed method approaches have also been found useful to better understand the level of each income and the perceptions and views of health workers on their different revenues [34]. Overall, it seems that the ideal approach would entail a combination of different methods, integrated into survey or interview tools that are practical and feasible to administer. Although collecting data on incomes routinely would be of high relevance for policy-makers, this possibility seems unlikely. As for the interpretation of results, while it may prove difficult to go beyond the context specificity of the findings of this type of research, cross-country comparisons may help to improve generalizability and find common patterns across contexts.

## Conclusion

In this paper, we have argued for an increased attention to the wider financial incentive environment and a better understanding of the complex remuneration of health workers, its determinants and the factors that underlie it, as well as its wide-ranging consequences for behaviour and performance. As recognized in the introduction, our perspective is limited because of its exclusive focus on financial incentives. In fact, we recognize that effective HRH strategies consist of “bundles of interventions”, which incorporate both financial and non-financial incentives, and our proposition does not aim to underestimate the importance of other non-financial motivation strategies. However, precisely because financial and non-financial incentives are complexly interrelated, remuneration is an essential element of any HRH strategy. It cannot be fully taken into consideration for policy-making without exploring and understanding the overall complex remuneration of health workers and the role it plays in determining recruitment, retention and motivation, as well as, more broadly, the performance of health systems and the progress towards universal health care.
